# Damage of Guinea Pig Heart and Arteries by a Trioleate-Enriched Diet and of Cultured Cardiomyocytes by Oleic Acid

**DOI:** 10.1371/journal.pone.0009561

**Published:** 2010-03-08

**Authors:** Josef Krieglstein, Tobias Kewitz, Uwe Kirchhefer, Oliver Hofnagel, Gabriele Weißen-Plenz, Michael Reinbold, Susanne Klumpp

**Affiliations:** 1 Institut für Pharmazeutische und Medizinische Chemie, Westfaelische Wilhelms-Universitaet, Muenster, Germany; 2 Institut für Pharmakologie und Toxikologie, Universitaetsklinikum Muenster, Muenster, Germany; 3 Leibniz-Institut fuer Arterioskleroseforschung, Universitaetsklinikum Muenster, Muenster, Germany; Universität Würzburg, Germany

## Abstract

**Background:**

Mono-unsaturated fatty acids (MUFAs) like oleic acid have been shown to cause apoptosis of cultured endothelial cells by activating protein phosphatase type 2C α and β (PP2C). The question arises whether damage of endothelial or other cells could be observed in intact animals fed with a trioleate-enriched diet.

**Methodology/Principal Findings:**

Dunkin-Hartley guinea pigs were fed with a trioleate-enriched diet for 5 months. Advanced atherosclerotic changes of the aorta and the coronary arteries could not be seen but the arteries appeared in a pre-atherosclerotic stage of vascular remodelling. However, the weight and size of the hearts were lower than in controls and the number of apoptotic myocytes increased in the hearts of trioleate-fed animals. To confirm the idea that oleic acid may have caused this apoptosis by activation of PP2C, cultured cardiomyocytes from guinea pigs and mice were treated with various lipids. It was demonstrable that oleic acid dose-dependently caused apoptosis of cardiomyocytes from both species, yet, similar to previous experiments with cultured neurons and endothelial cells, stearic acid, elaidic acid and oleic acid methylester did not. The apoptotic effect caused by oleic acid was diminished when PP2C α and β were downregulated by siRNA showing that PP2C was causally involved in apoptosis caused by oleic acid.

**Conclusions/Significance:**

The glycerol trioleate diet given to guinea pigs for 5 months did not cause marked atherosclerosis but clearly damaged the hearts by activating PP2C α and β. The diet used with 24% (wt/wt) glycerol trioleate is not comparable to human diets. The detrimental role of MUFAs for guinea pig heart tissue *in vivo* is shown for the first time. Whether it is true for humans remains to be shown.

## Introduction

In previous work, protein phosphatase 2C α and β (PP2C) has been shown to be activated by mono-unsaturated fatty acids (MUFAs) with special structural properties such as cis-configuration, the double bond in the middle of the molecule, and at least 15 C-atoms in length [1]. This activation of PP2C causes apoptosis of various cells including neurons and astrocytes [2], endothelial cells [3]–[7] and macrophages [7]. However, the concentration of MUFAs activating PP2C has to be relatively high in the culture medium of the cells (50–250 µM) and the question arises whether those concentrations of MUFAs can be achieved in the cells *in vivo*. It seems doubtful that this could be the case in brain cells *in vivo* but it may happen in arterial vessel cells like endothelial cells and macrophages [7]. These cells are exposed to high plasma levels of free fatty acids due to the fact that lipoprotein lipase (LPL) hydrolyzes triglycerides from lipoproteins [8]. Accordingly, we treated cultured endothelial cells and macrophages by MUFAs liberated from VLDL and observed an increase in apoptosis [6]. High levels of the plasma lipoproteins LDL and VLDL are among the pathophysiologic stimuli that induce endothelial apoptosis [9], [10] leading to endothelial dysfunction [11], [12]. Finally, the disruption of the endothelium is the initial trigger for atherogenesis [13]. Therefore, the hypothesis arises that stimulating PP2C and subsequent damaging endothelial cells by MUFAs contribute to atherogenesis. To confirm this idea, we investigated the effect of MUFAs on arterial vessels *in vivo*. Thus, we fed guinea pigs with a trioleate-enriched diet for 5 months and looked for pathological changes of the aorta, coronary arteries and the heart tissue. The guinea pig seems to be a suitable animal model to demonstrate the influence of lipids *in vivo* [14] and is considered appropriate to study atherosclerosis [15]. In our experimental setting we found moderate atherosclerotic changes but no atherosclerotic plaques of the vessel walls. Instead, we detected severe changes of the cardiac tissue and could demonstrate apoptosis of cultured cardiomyocytes caused by oleic acid. Of course, the results obtained are only true for guinea pigs and are not transferable to humans without further research.

## Methods

### Ethics Statement

Animals were maintained and treated according to the guidelines of animal welfare. The investigation conforms with the Guide for the Care and Use of Laboratory Animals published by the US National Institutes of Health (NIH Publication No. 85-23, revised 1996). In addition, approvals of our investigation were granted by government institutions (Regierungspräsidium Gießen V54-19c20-15MR 16/4 Nr. 21/2004 and Landesamt für Natur, Umwelt und Verbraucherschutz Nordrhein-Westfalen 8.87-50.10.46.08.180).

### Animal Experiments

Dunkin-Hartley-Guinea pigs (7 weeks old, 350–400 g) were obtained from Charles River (Sulzfeld, Germany) and the diets were purchased from Altromin (Lage, Germany). The animals were randomized into two groups: a control group (n = 13) and a trioleate group (n = 14). Both groups were fed *ad libitum* for 150 d and maintained in air-conditioned animal facility (23°C, 55% air humidity) on a reverse 12∶12 h light-dark cycle. The control group was fed with a standard guinea pig diet (Altromin item no. C3056) and the trioleate-diet group were fed with a modified C3056 diet containing 24% (wt/wt) glycerol trioleate. Thereby, fat represented 59% of the total energy content of the trioleate-diet and 0.3% of the control diet. These diets had similar calories, and the intake of amino acids, vitamins, mineral nutrients and trace elements remained constant. Food was stored at −80°C to prevent oxidation of fatty acids.

Guinea pigs were sacrificed by intraperitoneal injection of 100 mg of pentobarbital. For histological studies the heart and the aortic samples were immediately embedded in cryoprotective medium (Thermo Shandon, Waltham, USA) and snap-frozen in liquid nitrogen for subsequent cryosectioning. Sections (10 µm) were placed on polylysine-coated slides (Menzel, Braunschweig, Germany). Tissue and slides were stored at −80°C.

### Determination of Lipids in Blood

Blood samples were taken by cardiac puncture from the sacrificed guinea pigs and centrifuged at 2000×g for 15 min. Serum was extracted and stored at −80°C. Triglycerides, serum total cholesterol, low-density lipoprotein (LDL) cholesterol and high-density lipoprotein (HDL) cholesterol contents were assayed using the cholesterol oxidase phenol 4-aminoantipyrine peroxidase (CHOD-PAP) method or the glycerol phosphate oxidase-p-aminophenazone (GPO-PAP) method.

### Adult Ventricular Cardiomyocytes from Guinea Pigs

The guinea pigs (cavia porcellus) for preparing primary culture of cardiomyocytes were obtained from Prof. Sachser (Institut fuer Neuro- und Verhaltensbiologie, Muenster). Animals were prepared at 3–4 weeks of age with a weight of 200–250 g and were killed by cervical dislocation. The heart was removed and mounted on a Langendorff system for retrograde perfusion at a constant flow rate of 6 ml/min with an oxygenated MOPS buffer (100 mM NaCl, 1.2 mM KH_2_PO_4_, 5 mM MgSO_4_, 50 mM taurine, 10 mM glucose, 10 mM MOPS, pH 6.9) at 37°C. After a washout period of 8–10 min, the heart was perfused with oxygenated MOPS buffer containing 0.1% collagenase Worthington type II (Biochrom, Berlin, Germany) for 25–30 min. The heart was then placed in oxygenated broth (70 mM KCl, 30 mM K_2_HPO_4_, 5 mM MgSO_4_, 20 mM taurine, 20 mM glucose, 7.3 mM succinate, 5 mM creatine, 1 mM ethylene glycol tetraacetic acid (EGTA), 5 mM ß-hydroxybutyrate, 5 mM ATP, pH 7.4) and the ventricles were cut and chopped with scissors. The minced ventricles were incubated for 1 h in the broth and cells were separated by filtering through a nylon gauze filter with a pore size of 200 µm. The Ca^2+^-concentration was raised to 0.88 mM in five steps. Subsequently, cells were centrifuged for 3 min at 25 g and the sediment was resuspended in M199 medium (Gibco-Invitrogen, Karlsruhe, Germany) containing 2% FCS (PAA, Pasching, Austria), penicillin/streptomycin (100 U/ml; 100 µg/ml, Gibco-Invitrogen, Karlsruhe, Germany) and amphotericin B (0.5 µg/ml, Gibco-Invitrogen, Karlsruhe, Germany). Before the cells were seeded, the well plates (24-well) were preincubated for 3 h with M199 medium containing 2% FCS, penicillin/streptomycin and amphotericin B under 5% CO_2_ at 37°C. After the incubation the medium was removed and 1 ml of the cell suspension (5×10^5^ cells/ml) was then plated into each well and the cells were cultured at 37°C under an atmosphere consisting of 5% CO_2_ and 95% air. After the attachment period the medium was changed again and the cardiomyocytes were then treated for 24 h with oleic acid, stearic acid, elaidic acid or oleic acid methylester (Sigma Aldrich, Taufkirchen, Germany) at the concentrations indicated. The lipids were dissolved in DMSO, and DMSO was used as control. The final concentration of DMSO in the culture medium did not exceed 0.1%.

### Postnatal Ventricular Cardiomyocytes from Mice

Primary neonatal cardiomyocytes from mice (FVB/N strain) were prepared and maintained as described [16]. The cells were cultured in 24-well-plates with DMEM (Gibco-Invitrogen, Karlsruhe, Germany) containing 2% FCS and penicillin/streptomycin. The cardiomyocytes were treated for 24 h with oleic acid, stearic acid, elaidic acid or oleic acid methylester at the concentrations indicated.

### Western Blotting

Cardiomyocytes were harvested with a lysis buffer containing 10% glycerol, 3% SDS, 1 mM phenylmethanesulphonylfluoride, 1 µM calpain inhibitor, and 7 µg/ml trypsin inhibitor. Cell lysate proteins were analyzed on 15% SDS-PAGE and transferred to nitrocellulose membranes. Blots were blocked for 1 h at room temperature (RT) with 5% skim milk powder in Tris buffered saline supplemented with 0.1% Tween (TBS-T), incubated overnight at 4°C in TBS-T containing 0.1% BSA and primary antibody anti-rabbit PP2Cα (1∶100) [17] or anti-rabbit PP2Cß (1∶100) [17], or TBS-T containing 5% skim milk powder and anti-mouse α-tubulin (1∶10.000, Sigma Aldrich, Taufkirchen, Germany), followed by incubation in TBS-T for 1 h with peroxidase-conjugated anti-mouse or anti-rabbit secondary antibody (1∶2500, GE Healthcare, Chalfont St. Giles, United Kingdom). Blots were developed with ECL reagent (Thermo Fisher Scientific, Waltham, USA).

### Staining with Hoechst 33258 and Nile Blue

Hoechst 33258 (Sigma Aldrich, Taufkirchen, Germany) staining was used to detect apoptotic cardiomyocytes. After the treatment with the lipid for 24 h, the cells were washed with PBS (phosphate buffered saline, pH 7.4), fixed for 30 min with paraformaldehyde (4%) and then incubated for 30 min with the DNA fluorochrome Hoechst 33258 (10 µg/ml) in methanol at RT in the dark. After washing with PBS, the nuclear morphology was analyzed under a fluorescent microscope (Axiovert 100, Zeiss, Jena, Germany) at an excitation wavelength of 350 nm and an emission wavelength of 450 nm. Cells showing shrunken or fragmented nuclei or chromatin condensation were counted as apoptotic cells. Nile blue (Sigma Aldrich, Taufkirchen, Germany) was used to demonstrate lipid uptake into cardiomyocytes. After staining with Hoechst 33258, cardiomyocytes were washed with PBS, stained with Nile blue solution (10 µg/ml) for 2 h and analyzed under a confocal laser scanning microscope (LM510 from Zeiss, Jena, Germany) at wavelengths of 488/525 nm.

### Immunocytochemistry

For immunocytochemistry, the cardiomyocytes were stained with Hoechst 33258 and afterwards the cell membrane was permeabilized by incubation with 0.2% Triton X-100 for 5 min, then cells were treated with 5% goat serum (detection of PP2C α and β) or 5% horse serum (detection of α-actinin) in PBS for 30 min. Cardiomyocytes were washed three times with PBS and subsequently incubated with anti-PP2C α and β antibody (1∶100) or anti-α-actinin (1∶300, Sigma Aldrich, Taufkirchen, Germany) overnight at 4°C, respectively. For the detection of PP2C α and ß, cells were washed the following day with PBS, incubated with a biotinylated secondary antibody (1∶200, Vector Laboratories, Burlingame, USA) for 1 h at RT and then incubated with Fluorescein Avidin D (Vector Laboratories, Burlingame, USA) for 1 h in darkness. For the detection of α-actinin, the cells were washed the following day with PBS and then incubated with the Texas Red conjugated antibody (1∶200, Vector Laboratories, Burlingame, USA) for 1 h at RT. Finally, the cells were analyzed under a laser scanning-microscope (LM510 from Zeiss, Jena, Germany).

### Knock-Down of PP2C in Cardiomyocytes

To induce RNA interference (RNAi), primary mice neonatal cardiomyocytes were transfected with small interfering RNA (siRNA) oligonucleotides directed against mRNA of PP2C α and PP2C β. The siRNA was designed as 21mers according to published guidelines, and had dTdT 3′-overhangs [18]. For knock-down of PP2Cα, 5′-GUA CCU GGA GAG CAG AGU A-3′, corresponding to the mRNA target sequence 5′-AAG TAC CTG GAG AGC AGA GTA-3′ and for PP2Cβ, 5′-UGU UAU UGA AGC UGU UUA U-3′, corresponding to 5′-AAU GUU AUU GAA GCU GUU UAU-3′ were generated. Cells were prepared as described [16] and were grown in a 1∶5 mixture of M199/DMEM with 1% horse serum. Transfection was carried out at the third day after isolation using 4 µl/ml of oligofectamine and 100 nmol of siRNA. Oligofectamine and siRNA were separately diluted in M199/DMEM medium without horse serum, mixed and incubated for 30 min at RT. Sixteen hours after the onset of transfection, non-transfected as well as transfected cardiomyocytes were treated with 200 µM oleic acid for additional 4 h. Control cells were incubated with medium plus oligofectamine. The primary cultures used contained 60–70% cardiomyocytes and 30–40% fibroblasts. Cardiomyocytes were identified by α-actinin.

### DNA Nick End Labeling of Heart Sections

The presence of nuclear DNA fragmentation, a marker of cell death, was assessed in cross sections of the ventricles (maximal ventricular diameter) with the use of the Dead End Colorimetric TUNEL System (Promega, Mannheim, Germany). This system measures the fragmented DNA of apoptotic cells by incorporating biotinylated nucleotides at 3′-OH DNA ends using the recombinant terminal deoxynucleotidyl transferase (rTdT). The horseradish peroxidase-labeled streptavidin (HRP-SA) is then bound to these biotinylated nucleotides and HRP-SA detected using H_2_O_2_ and the chromogen diaminobenzidine. Apoptotic nuclei are stained dark brown. Controls included incubations in the presence of deoxyribonuclease I (positive control) and incubations without rTdT (negative control). TUNEL-positive cells were counted at a magnification of 20 and given as the number per mm^2^.

### Staining with Haematoxylin-Eosin and Oil Red O

Aorta and heart sections were analyzed by haematoxylin-eosin (HE) and oil red O-staining. For general histology HE-staining was performed. The slides were dried for 20 min at RT, fixed with paraformaldehyde (4%) for 15 min, washed twice with PBS for 5 min and once with distilled water for 5 min. The tissue sections were stained with Mayers hemalaun (Chroma, Muenster, Germany) for 5 min, washed in tap water for 10 min, immersed in eosin solution (0.5% eosin (Chroma, Muenster, Germany), 1% acetic acid, in distilled water) for 3 min and washed with distilled water. The slides were then dehydrated in 70% ethanol (3 min), 96% ethanol (3 min), 99% ethanol (2×3 min), xylol (2×3 min) and were mounted in entellane (Merck, Darmstadt, Germany) and finally covered with a coverslip.

The lipid deposition in the sections was demonstrated with oil red O staining. The slides were dried for 20 min at RT, fixed with paraformaldehyde (4%) for 15 min and then washed with PBS three times for 5 min. The tissue sections were incubated with oil red O solution (0.3 g oil red O (Sigma Aldrich, Taufkirchen, Germany) in 100 ml isopropanol) for 10 min, washed in distilled water, mounted in Dako Fluorescent Mounting Medium (Dako Cytomation, Glostrup, Denmark) and covered with a coverslip.

### Protein Determination

The concentration of proteins was determined by Lowry-assay [19] using bovine serum albumin as a standard.

### Statistics

All values are given as means ± SD. One way analysis of variance (ANOVA) followed by Scheffé's test was applied.

## Results

### Dietary Uptake and Plasma Lipid Composition of the Guinea Pigs

**Figure 1:**
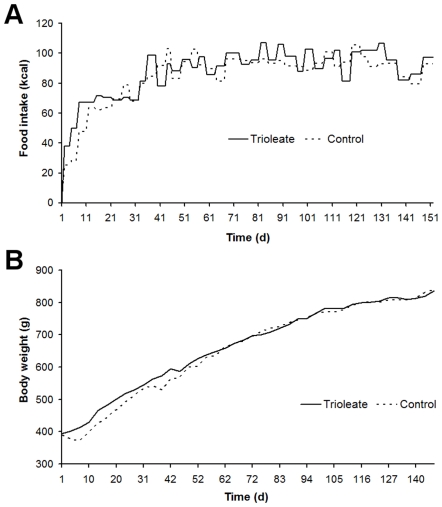
Food consumption and development of the weight of the guinea pigs. (A) Food uptake measured in calories and (B) body weight in gram of the guinea pigs from both groups.

Both diets were well tolerated by the guinea pigs. The average daily energy consumption of 90–100 kcal did not differ significantly between the trioleate-fed animals and the controls (Fig. 1A) and the increase of the body weight was similar in both groups (Fig. 1B).

**Figure 2:**
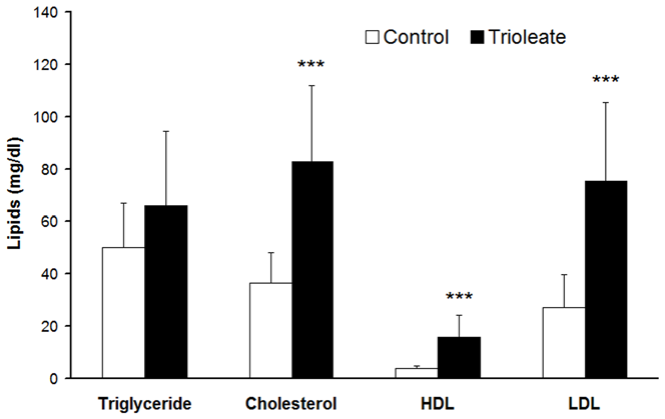
Levels of the serum lipids after feeding a trioleate-enriched diet for 5 months. Total cholesterol, LDL cholesterol and HDL cholesterol, but not triglycerides, were significantly increased in the serum of the trioleate-fed guinea pigs. Serum lipids (mg/dl) are given as means ± SD. ***p<0.001, compared with control group.

The serum concentration of the triglycerides was not significantly affected by the oleic acid content of the diet, but the concentration of total cholesterol (82.66±29.06 mg/dl), LDL cholesterol (75.31±30.00 mg/dl) and HDL cholesterol (15.90±8.38 mg/dl) were significantly higher in the trioleate-diet group compared with the control diet group (total cholesterol 36.63±11.89 mg/dl, LDL cholesterol 27.19±12.03 mg/dl and HDL cholesterol 3.76±1.01 mg/dl) (Fig. 2).

### Atherosclerotic Lesions in the Aortas

**Figure 3:**
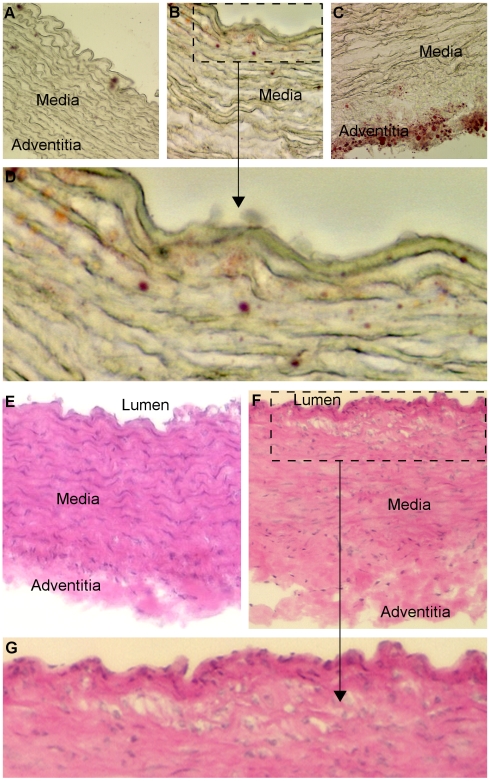
Trioleate-induced changes of the aorta. Oil red O staining: The aortas from the control diet guinea pigs had no fat inclusions (A). In contrast, the aortas from the trioleate-fed guinea pigs showed some lipid depositions in the subendothelium (B, D) and in the adventitia (C). Furthermore, focal areas with elastin degradation and disorganisation were detected (B, C). HE-staining: The aortas from the control group exhibited a compact structure (E). The media consisted of homogenous layers of thick elastic lamellae and smooth muscle cells. The adventitia was composed of compact connective tissue with interspersed adventitial cells. In comparison, the aortas from the trioleate group exhibited deterioration of the smooth muscle cells and of structural integrity in the media (F. G). The connective tissue of the adventitia was spongy and fewer cells were observed (F). Particularly, in the subendothelial area clusters of foamy smooth muscle cells were found (G). A, C  =  original magnification ×20; B, E, F  =  original magnification ×40.

The aortas from the control group were devoid of lipid staining (Fig. 3A). After trioleate diet, some lipid depositions were detected in the subendothelium (Fig. 3B, D) and in the adventitia (Fig. 3C). In addition, the aortas from the trioleate group exhibited focal areas with elastin degradation and disorganisation (Fig. 3B, C). HE-staining of the aortas from the control group exhibited a compact structure (Fig. 3E). The media consisted of homogenous layers of thick elastic lamellae and smooth muscle cells. The adventitia was composed of a compact connective tissue layer with interspersed adventitial cells. In comparison, the aortas from the trioleate group showed disintegrations of the smooth muscle cells and elastic lamellae in the media. The connective tissue of the adventitia was spongy and less cells than in controls were observed (Fig. 3F). Particularly, in the subendothelial area clusters of foamy smooth muscle cells were found (Fig. 3G).

### Damaging Effects of the Trioleate Diet on the Heart

**Figure 4:**
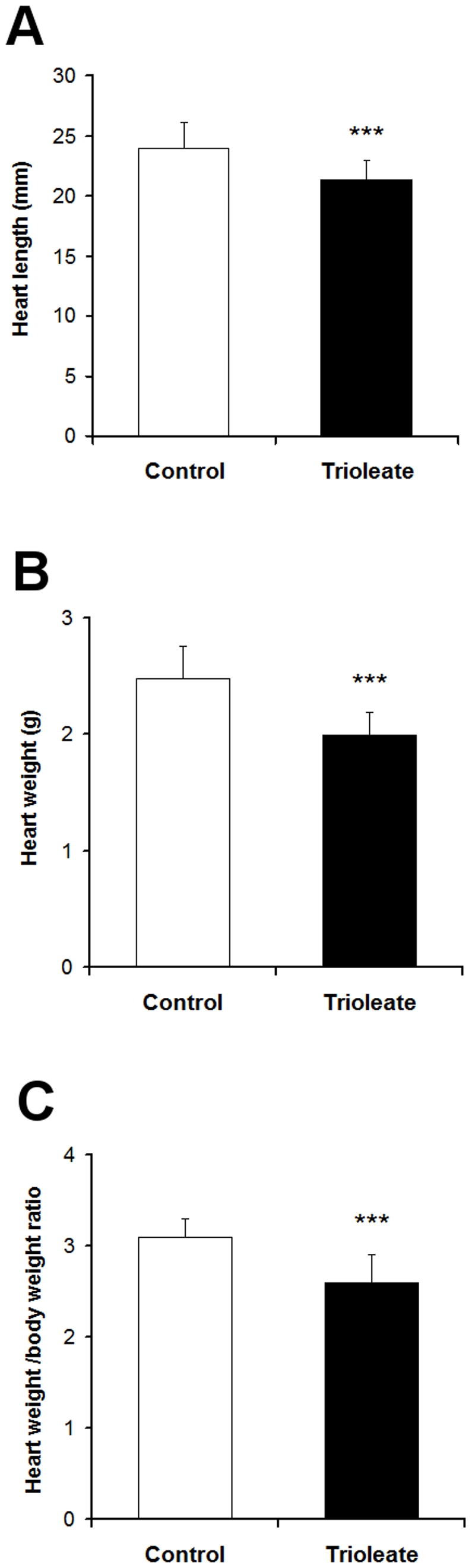
Size and weight of the guinea pig hearts. The trioleate-fed guinea pigs show a significantly decreased length (A) and weight (B) of the heart. The ratio of heart weight to body weight (g/kg) was significantly decreased in the trioleate-fed guinea pigs (C). Values are given as means ± SD. ***p<0.001, compared with control group.

**Figure 5:**
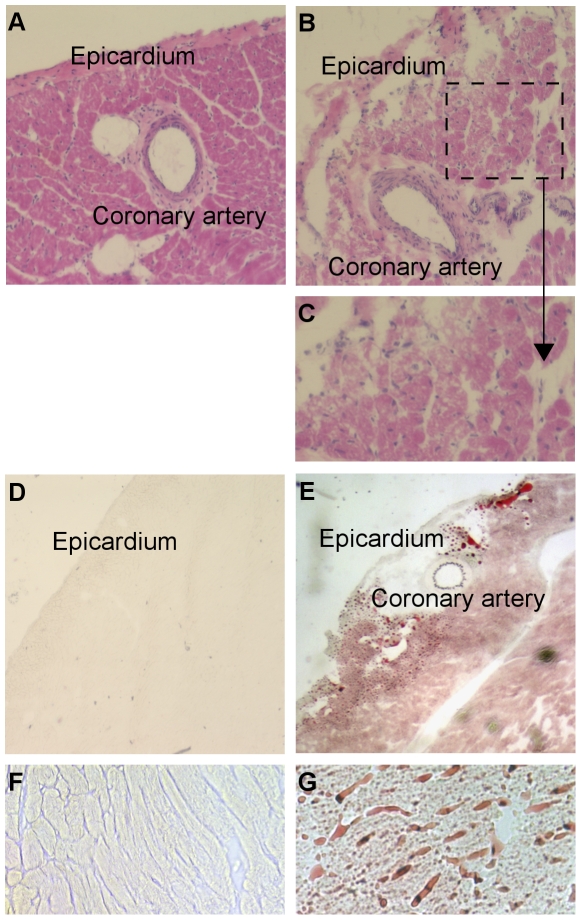
Trioleate-induced damage of guinea pig hearts. The HE-staining visualized a compact structure of the myocardium of the control-diet group (A). In contrast, trioleate-diet resulted in perivascular fibrosis and a structural deterioration of the myocyte compartment (B, C). Numerous myocytes appeared foamy (B, C). In the coronary arteries of both groups intimal thickening was not observed (A, B, E). Oil red O staining: Lipid accumulations were not found in the control hearts (D, F). In comparison, lipids accumulated throughout the entire myocardium of the trioleate-diet group (E, G). Although massive lipid deposits were detected in the myocardial areas adjacent to coronary arteries, mural lipid deposits were not observed (E). Moreover, lipids accumulated in the interstitial space (G). A, B  =  original magnification ×10; D, E  =  original magnification ×2,5; F, G  =  original magnification ×20.

The trioleate-fed guinea pigs showed a significantly decreased length and weight of the hearts (Fig. 4A, B). Also, the ratio of heart weight to body weight (g/kg) was decreased significantly in the trioleate-fed guinea pigs (Fig. 4C). General histology of the control myocardium revealed a compact structure of the myocyte compartment and the interstitial tissue (Fig. 5A). In comparison, myocytes of the trioleate group showed a loss of structural integrity and numerous myocytes appeared foamy (Fig. 5B, C). In the coronary arteries of both groups intimal thickening was not observed (Fig. 5A, B). Lipid depositions were not found in the control hearts (Fig. 5D, F). In comparison, lipids accumulated throughout the entire myocardium of the trioleate-diet group (Fig. 5E, G). Although massive lipid deposits were detected in the myocardial areas adjacent to coronary arteries, mural lipid deposits were not observed (Fig. 5E). In addition, lipids accumulated in the interstitial space (Fig. 5G). Significantly more apoptotic muscle cells were detected by TUNEL staining in the hearts from trioleate-fed animals (4.4±1.8 cells/mm^2^, n = 14) compared to the hearts from control animals (1.3±1.1 cells/mm^2^, n = 13; means ± SD; p<0.001).

### Induction of Apoptosis in Cultured Cardiomyocytes

**Figure 6:**
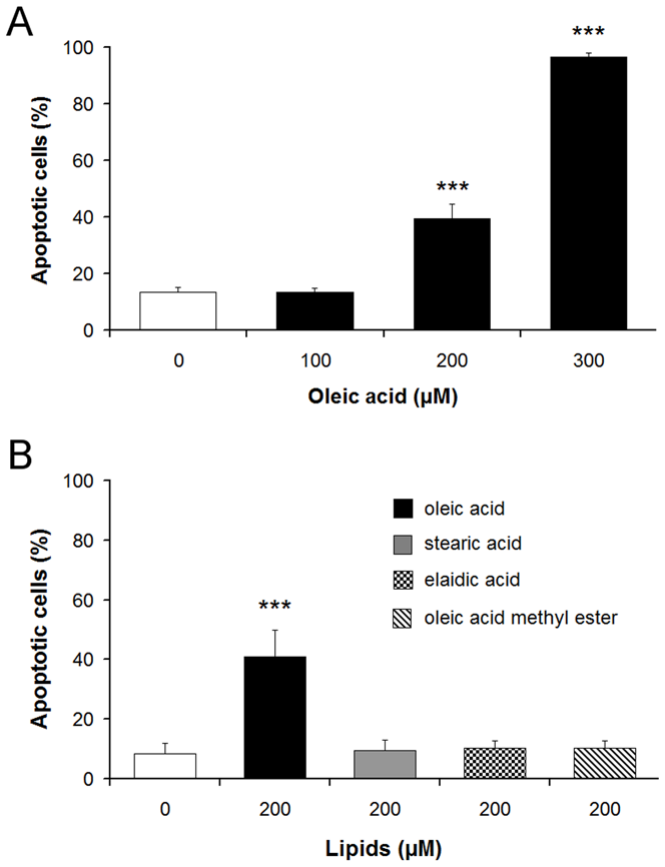
Oleic acid but not stearic acid, elaidic acid or oleic acid methylester damage cardiomyocytes. Guinea pig cardiomyocytes were incubated with different concentrations of oleic acid and oleic acid induced apoptosis in a dose-dependent manner (A). In contrast, stearic acid, elaidic acid or oleic acid methylester in the concentration range of 200 µM did not stimulate apoptosis (B). Control cardiomyocytes were treated with vehicle only (0.1% DMSO). Apoptotic cells are expressed as percentage of the total number of cells. Means ± SD of 6 experiments are given. ***p<0.001, compared with vehicle-treated control.

**Figure 7:**
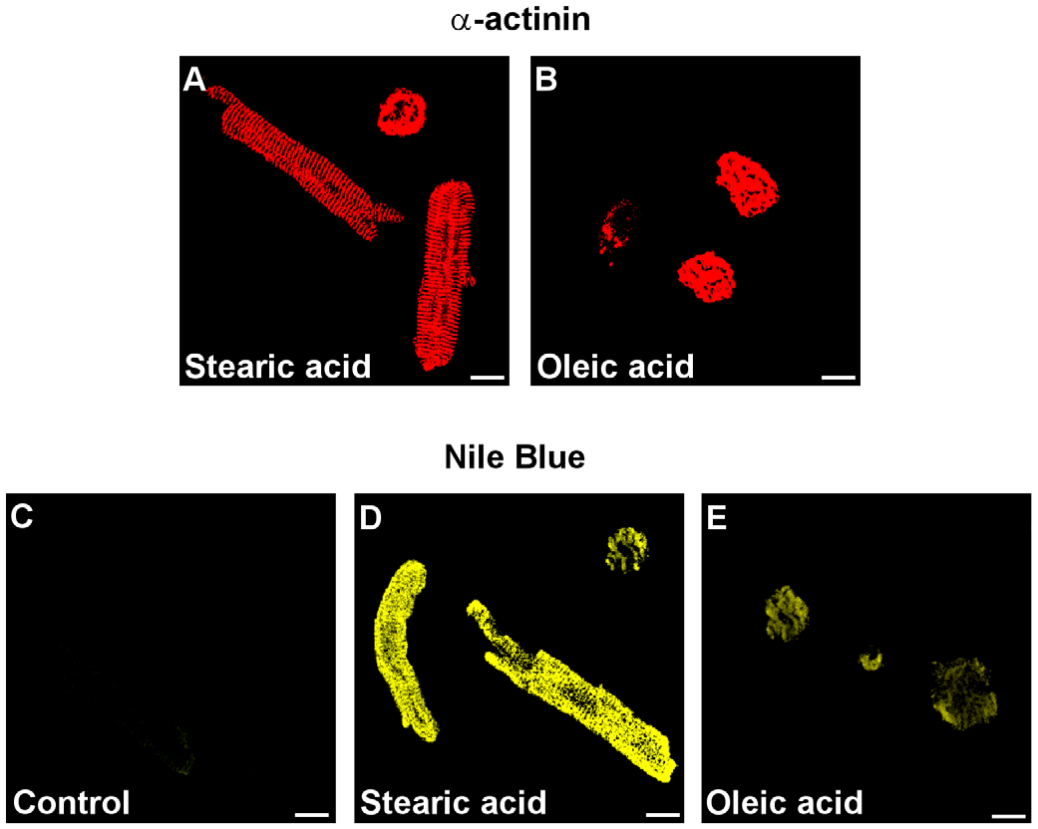
Uptake of fatty acids into guinea pig cardiomyocytes. Guinea pig cardiomyocytes were incubated for 24 h with 200 µM stearic acid (A, D) or 200 µM oleic acid (B, E) and were identified with α-actinin (A, B). Additionally, Nile blue staining showed that both fatty acids were taken up by the cells but, notably, oleic acid-treated cells were partially damaged and stearic acid-treated were not. –  = 10 µm.

To verify the damaging role of the oleic acid-activated PP2C in the heart tissue, it was necessary to explore oleic acid-induced apoptosis in primary cultures of guinea pig cardiomyocytes. It was shown that oleic acid stimulated the activity of PP2C, whereas the oleic acid-derivatives elaidic acid and oleic acid methylester did not [1], [2]. In general, the structural requirements for activation of PP2C strikingly correlated with the induction of apoptosis in neurons and endothelial cells [2], [3]. To evaluate this relationship in cells of the heart muscle, cultured cardiomyocytes from guinea pigs were treated with oleic acid, stearic acid, elaidic acid or oleic acid methylester. Exposure of cardiomyocytes to oleic acid induced apoptosis in a dose-dependent manner (Fig. 6A). In contrast, the presence of stearic acid, elaidic acid or oleic acid methylester at a concentration of 200 µM did not cause apoptosis in cardiomyocytes (Fig. 6B). Immunolocalization of α-actinin in fatty acid-treated cells demonstrated the typical structure of cardiac myofibrils. Oleic acid-treated cardiomyocytes (Fig. 7B) revealed apoptosis whereas stearic acid-treated cells (Fig. 7A) remained intact. By staining the cardiomyocytes with Nile blue we could demonstrate that both oleic acid and stearic acid were taken up into the cells at a similar extent but only oleic acid caused apoptosis (Fig. 7C–E). Hence, the cell death cannot be induced simply by lipid overload.

**Figure 8:**
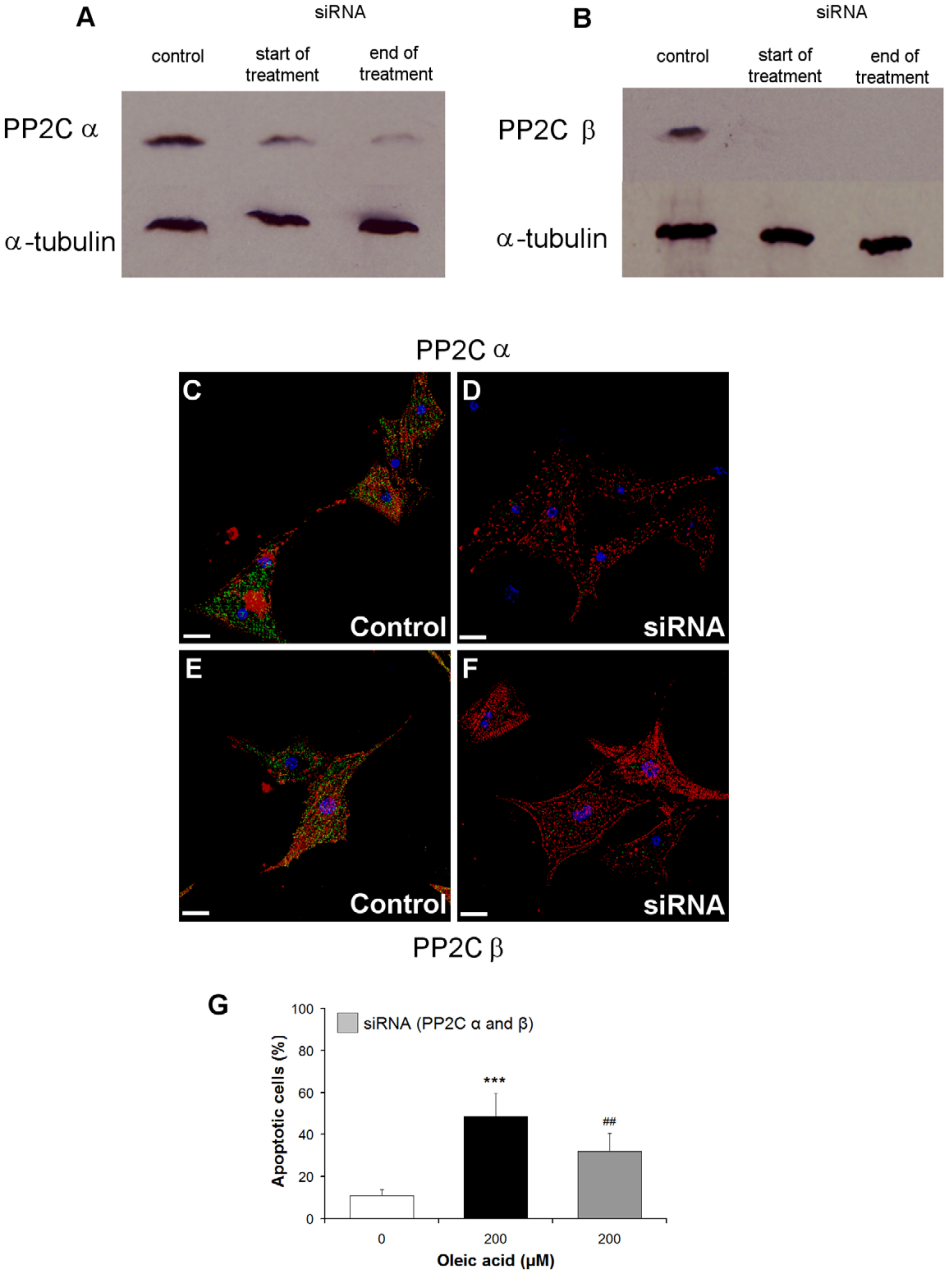
PP2C is causally involved in oleic acid-induced apoptosis in neonatal cardiomyocytes from mice. Sixteen hours after the onset of transfection, non-transfected as well as transfected cardiomyocytes were treated with 200 µM oleic acid for 4 h. Control cells were incubated with medium plus oligofectamine. PP2C α and β were analyzed by western blot at the beginning and at the end of the experiment (A, B). At both time points, the enzymes in the transfected cells were reduced compared to non-transfected cells. α-Actinin staining (red) demonstrated the typical structure of neonatal cardiomyocytes (C–F). Downregulation of PP2C α and β (green) was observed in these cells (D, F). The cardiomyocytes with reduced PP2C levels were less damaged by oleic acid (G). Apoptotic cells are expressed as percentage of the total number of cells ± SD. ***p<0.001, compared with vehicle-treated control, ^##^ *p*<0.01, compared with non-transfected cells treated with oleic acid. −  = 10 µm.

To verify the involvement of PP2C in oleic acid-induced apoptosis in cardiomyocytes we reduced the synthesis of PP2C α and β simultaneously in the cardiomyocytes as both of them can be activated by oleic acid. Oleic acid treated-cells with lower levels of the enzymes should be less damaged. It was not possible to knock-down the PP2C isoforms in guinea pig cardiomyocytes because the cells did not survive the combination of transfection with serum deprivation. Therefore, we used neonatal mouse cardiomyocytes to knock-down PP2C. Similar to cardiomyocytes from guinea pig oleic acid but not stearic acid, elaidic acid or oleic acid methylester induced apoptosis in cardiomyocytes from mice (data not shown). The primary cultures of cardiomyocytes contained 30–40% fibroblasts. Because PP2C α and β were present in both cardiomyocytes and fibroblasts (data not shown), PP2C α and β were downregulated in both cell types. The amount of PP2C α and β was analyzed at the beginning and the end of the oleic acid treatment. At both time points, the enzyme protein content in the transfected cells was reduced compared to non-transfected cells (Fig. 8A, B). In addition, α-actinin staining demonstrated the typical structure of postnatal cardiomyocytes (Fig. 8C–F) and also showed downregulation of PP2C α and β (Fig. 8D, F). The cardiomyocytes with reduced PP2C levels were less damaged by oleic acid (Fig. 8G), demonstrating lower activity of PP2C and subsequently reduced apoptosis.

## Discussion

During the last decade, we obtained strong evidence that MUFAs activate PP2C and the activated enzyme causes apoptosis in various cell types [3], [5]. Endothelial cells are susceptible to high levels of lipids. LPL on the luminal surface of endothelial cells hydrolyzes triglycerides and free fatty acids are taken up by the cells [20]. In a first approach we treated HUVECs with lipoproteins and initiated apoptosis when lipoproteins were cleaved by LPL [3]. We could also show that a physiological amount of LPL, produced by macrophages, was capable of liberating MUFAs at a concentration high enough to cause apoptosis in endothelial cells [6]. These results fostered the idea that apoptosis of endothelial cells caused by MUFAs could represent an initial step in the development of atherosclerosis. Therefore, we made an attempt to find out whether this apoptotic effect of oleic acid on endothelial cells and other cell types also is true in intact animals in vivo. We used guinea pigs for these experiments and fed them for 5 months with a diet enriched with glycerol trioleate. It is known that rodents like rabbits and guinea pigs develop lipid-rich arterial lesions with some of the features of atherosclerosis only if they are fed large amounts of cholesterol and fat, components that are usually lacking in their vegetarian diet [21]. Therefore, in our study guinea pigs were fed with diet containing 24% glycerol trioleate, i.e. 59% of the total energy content, whereas in most human diets about 30–40% of total dietary energy intake is provided by fat [22]. Nevertheless, the food intake, measured in calories, of the trioleate-fed animals was similar to that of controls and also the increase in the body weight was similar in both groups (Fig. 1). Thus, we can exclude that simply an altered food intake may influence our results. Interestingly, triglycerides did not significantly increase in the blood of the trioleate-fed animals, whereas cholesterol, HDL and LDL levels were significantly elevated (Fig. 2). However, the mean level of cholesterol was still around 80 mg/dl, a blood level of cholesterol which should not cause atherosclerosis and, in addition, the LDL/HDL ratio was low. Cos et al. [23] induced atherosclerotic plaques in guinea pigs by feeding the animals with dietary cholesterol in the range of 2000 mg/d for 12 weeks. Thereby, the animals had a serum concentration of 255 mg/dl cholesterol. The low cholesterol level in our experiments and the low LDL/HDL ratio might have been the reason why we did not find atherosclerotic plaques in the coronary arteries and the aortas. However, we found some lipid depositions and area clusters of foamy smooth muscle cells in the subendothelium (Fig. 3B, D, G). Therefore, it is suggested that trioleate diet may induce atherogenesis. Similar changes were found in the heart (Fig. 5). Fat droplets were seen around the coronary arteries and the myocardial tissue–including both the myocytes and the interstitial compartment–appeared slightly disintegrated. More apoptotic muscle cells were found by TUNEL staining in the trioleate-fed animals compared to control animals and this increase of cellular damage could be the reason for the decreased dimension and weight of the heart from the trioleate-fed animals (Fig. 4) but it cannot be excluded that also other parameters like a decreased blood pressure could have been responsible for that. The explanation for these results so far could be that the increased uptake of oleic acid did not cause atherosclerotic plaques despite the increased blood cholesterol level because the LDL/HDL ratio was decreased. However, oleic acid obviously diffused into heart tissue and into myocytes, stimulated PP2C and caused myocardial damage as demonstrated. These are exciting results but they are not allowed to be uncritically transferred to the situation in humans.

The study examined the novel hypothesis that an increased fat uptake caused damage of the heart by apoptosis of cardiomyocytes. To be sure that oleic acid is capable of inducing apoptosis in cardiomyocytes similar to endothelial cells and macrophages, we made an attempt to strengthen this evidence obtained from guinea pigs. To this end we established primary cultures of mouse and guinea pig cardiomyocytes. Similar to previous experiments with other cells like HUVECs or neurons, we treated the cultured cardiomyocytes with oleic acid, stearic and elaidic acid, and oleic acid methylester and we got similar results (Fig. 6). Oleic acid caused apoptosis in mouse and guinea pig cardiomyocytes but stearic and elaidic acid as well as the oleic acid methylester did not. To verify its role in apoptosis of cardiomyocytes PP2C expression was downregulated by siRNA (Fig. 8A, B). In these cells with lowered PP2C level the damaging effect of oleic acid was significantly diminished demonstrating that PP2C is causally involved (Fig. 8G). These results were in line with our earlier findings on endothelial cells and macrophages but also on neurons and astrocytes [3], [4], [7]. Since PP2C is a phosphatase which is distributed ubiquitously in the mammalian organism [24], [25] high amounts of MUFAs may cause apoptosis of every cell provided that the concentration of MUFAs is high enough. The point is that such high levels of MUFAs cannot be achieved in each type of cells. However, it seems to be easily possible in endothelial cells. In addition, as shown in the present paper a high fat uptake may also increase the level of MUFAs in cardiac tissue, high enough to activate PP2C and damage muscle cells.

In conclusion, because of the anti-atherogenic effect of the decreased LDL/HDL ratio under the trioleate-enriched diet, guinea pigs did not develop atherosclerotic plaques but the arteries remained in a pre-atherosclerotic stage of vascular remodelling. However, lipids accumulated in the heart and damaged heart tissue. Experiments with cultured cardiomyocytes confirmed the idea that MUFAs activated PP2C and subsequently caused apoptosis. This study reveals for the first time the detrimental role of MUFAs for the heart. On the other hand, there is a large body of evidence showing a favourable role of MUFAs in human health. For instance, a reduced rate of coronary heart disease in traditional Mediterranean populations has been reported by Keys [26] and Mensink et al. [27] demonstrated in a meta-analysis that the atherosclerotic risk was diminished by MUFAs, but it cannot be neglected that the detrimental effects of MUFAs on various cells in culture and in intact animals mediated by activation of PP2C are well documented. It remains to be shown which role these effects play in humans.
